# Innovative Blown Multi-Micro-Nano-Layer Coextrusion: Insights into Rheology and Process Stability

**DOI:** 10.3390/polym17010057

**Published:** 2024-12-29

**Authors:** Lazaros Vozikis, Skander Mani, Abderrahim Maazouz, Khalid Lamnawar

**Affiliations:** 1 CNRS, UMR 5223, Ingénierie des Matériaux Polymères, INSA Lyon, Université de Lyon, F-69621 Villeurbanne, France; lazaros.vozikis@insa-lyon.fr (L.V.); abderrahim.maazouz@insa-lyon.fr (A.M.); 2 CT-IPC, 2 Rue Pierre et Marie Curie, F-01100 Bellignat, France; skander.mani@ct-ipc.com

**Keywords:** blown coextrusion, multi-micro-nano-layer films, morphology, shear and extensional rheology, stability investigation

## Abstract

The present study introduces an innovative blown coextrusion die technology designed to address a critical gap in the production of multilayer films. Unlike conventional systems, this novel die allows for the creation of films with a high number of layers, ensuring layer integrity even in the micro-nano scale. A key advancement of this die is its ability to increase the number of layers without extending the residence time since it does not require an additional multiplier element. The risk of thermal degradation can, thus be, minimized. The die can easily be combined with existing cast coextrusion technologies, making it very versatile. Stability maps were developed to define processability and, in association with rheological analysis, optimal processing windows were determined. This study highlights the potential of enhancing material efficiency by increasing the number of layers while reducing the need for high percentages of EVOH. The produced multilayer films exhibited strong layer adhesion without the use of tie layers, thus improving recyclability and supporting sustainability goals.

## 1. Introduction

The growing demand for high-performance packaging materials has led to extensive research in the field of multilayer films. The forced-assembly multilayer coextrusion process represents an elaborate technology for the manufacture of multilayer films. This method was preliminarily developed by Dow Company and provides an exceptional possibility to combine multiple polymers into layers to create a final object. Each element of the multilayer structure can deliver its own end-use features, for instance, mechanical [1,2,3], barrier [4,5],dielectric [6,7], and optical properties [8,9,10]. It is being increasingly used in automotive engineering, smart materials, domestic appliances, and packaging materials [11,12]. Within these applications, ethylene-vinyl alcohol (EVOH) is highly valued for its gas barrier performance, making it a necessity in industries producing food packaging, pharmaceuticals, and other high-barrier applications [13,14].

While a large amount of research on cast coextrusion already exists, blown coextrusion remains underexplored, especially for the production of films with a high number of layers. Achieving micro-nano-scale layer thicknesses, while maintaining layer integrity, has been especially difficult. Traditional spiral mandrel and pancake-style dies face particular challenges in maintaining temperature control and uniform flow. Spiral mandrel dies are commonly employed but are limited in the coextrusion of multilayer films, mainly those with a high number of layers. Increasing the die diameter to accommodate more layers exacerbates residence time and increases the risk of thermal degradation [15]. On the other hand, pancake-style dies can provide better control over layer distribution but are prone to greater pressure drops, limiting their effectiveness when it comes to producing multilayer films with a high number of layers [12,16].

Recent advancements have addressed some of these challenges. Dooley et al. (2016) demonstrated the production of microlayer blown films with over 100 layers by combining a feedblock, multiplier elements, an encapsulation die, and a crosshead die [17]. Steinmetz et al. (2023) integrated multilayer coextrusion with blow molding to fabricate structures comprising hundreds or even thousands of layers, emphasizing scalability and structural integrity [18]. Hong et al. (2022) introduced a novel blown film technology that incorporated a custom-designed blown die with layer multiplication principles adapted from cast coextrusion. This method enabled the production of blown films with over 1000 layers and thicknesses below 10 μm, representing a breakthrough in nano-layer film technology [19]. These advancements highlight the promise of advanced coextrusion techniques and die designs in addressing the mentioned challenges, paving the way for innovation and further development in the field.

In response to these challenges, the present study introduces a novel blown coextrusion die technology, integrating multiplier elements and modular architectures to significantly increase the number of layers without a corresponding increase in residence time. These innovations not only enable the production of films with hundreds or even thousands of layers, but also improve process stability. The novel modular blown coextrusion die represents a promising solution, addressing many of the limitations encountered by conventional die technologies.

A key part of this study was exploring the potential of using forced assembly coextrusion with the novel blown die to enhance the barrier performance of multilayer films without increasing the amount of ethylene vinyl alcohol (EVOH). A detailed analysis of barrier properties will be covered in future work. Although this strategy has been widely studied and validated in cast coextrusion processes, mainly due to confinement or tortuosity phenomena, its application to blown film coextrusion has been limited [20,21,22,23,24]. In addition, the need for tie layers to ensure adhesion between layers is eliminated, further improving the recyclability of the films. This is particularly important for the circular economy, as it simplifies the recycling process by reducing the complexity of material synthesis [25].

This research has also addressed the critical issue of process stability by developing stability maps and performing in-depth rheological analyses. The first steps involved assessing flow stability through cast coextrusion, where challenges are often compounded when coextruding polymers with different rheological properties. Variations in viscosity or elasticity between layers can lead to instabilities, which significantly reduce film performance [26,27,28,29,30]. Following the evaluation of the processability window via cast coextrusion, the present study provides a comprehensive analysis of the parameters required to ensure stability during blown coextrusion. Critical processing parameters such as the blow up ratio (BUR) and take up ratio (TUR) play a crucial role in obtaining stable and defect-free multilayer films. Various instabilities can occur during the process, including drawing resonance, helical instability, frost line oscillation, bubble tearing, and bubble breathing, affecting both the process and final film quality. These instabilities are affected by a combination of factors, including processing conditions, cooling, and material properties [28,29,30,31,32,33,34,35,36,37].

In summary, this study presents a significant advancement in the field of blown coextrusion by introducing a novel technology that enables the production of films with improved barrier properties and sustainability. By focusing on reducing material costs and improving recyclability, the present work paves the way for next-generation packaging materials that are more efficient and environmentally friendly.

## 2. Materials and Methods

### 2.1. Materials

The materials used in this study were the constituents of multilayer films produced through the coextrusion process. The multilayer had a three-layer structure, where the external layer was composed of linear low density polyethylene (LLDPE), and the internal barrier layer was made from ethylene-vinyl alcohol copolymer (EVOH). The EVOH used had an ethylene content of 32 mol%. The specific details of each polymer, including density, melt flow rate (MFR), and manufacturer, are summarized in Table 1.

### 2.2. Characterization Techniques

#### 2.2.1. Rheological Study

Rheological measurements were carried out using a stress-controlled D-HR2 Discovery Hybrid Rheometer (TA Instruments, New Castle, DE, USA). The tests were performed in the linear viscoelastic region (LVR). The samples were prepared by compressing thin plaques at 180 °C for PE and 185 °C for EVOH. These plaques were then cut into 25 mm diameter discs and dried overnight under vacuum.

A MALVERN capillary rheometer (TA Instruments, New Castle, DE, USA) with a 180° entry angle die and L/D ratios of 30, 20, and 10 was used for the high shear measurements. The Bagley correction was obtained and the elongational viscosity was determined using the Cogswell model [38].

Additionally, uniaxial elongation tests were conducted using a SER2 (Sentmanat Extensional Rheometer, TA Instruments, New Castle, DE, USA) mounted on the DHR-2. Samples were prepared in the form of 0.5 mm thick, 10 mm wide, and 20 mm long rectangles.

To ensure reproducibility, all tests were performed five times under the same conditions.

#### 2.2.2. Morphological Characterization

A morphological characterization of the multilayer films was carried out using Transmission Electron Microscopy (TEM) to investigate the layer integrity and thickness distribution. The TEM analysis was performed using a JEOL 1400 Flash microscope (JEOL Ltd., Tokyo, Japan) on ultrathin sections (~80 nm) prepared by cryo-ultramicrotomy, with the sections cut perpendicular to the extrusion direction. 

### 2.3. Coextrusion of Multilayer Films

#### 2.3.1. Cast Coextrusion Setup for Flow Stability Investigation

A laboratory-built cast coextrusion setup was used for the flow stability investigation and to determine the processability window of multilayer films. Three extruders were used in the process. The polymers were first melted and combined in the feedblock, which determined the layer configuration. Prior to reaching the die, the structure went through a series of multiplier elements, during which it was repeatedly split and recombined until the development of the final multilayer structure. The feedblock and the number of multiplier elements employed determined the total number of layers.

#### 2.3.2. Development and Design of the New Blown Coextrusion Die

Several challenges and requirements in blown film production led to the development of the new blown coextrusion die technology. While cast coextrusion has been widely studied, blown coextrusion is less presented in the literature. Our innovative die design bridges this gap by providing a robust and adaptable solution that enhances blown coextrusion capabilities, enabling the precise formation of multilayer films with superior layer integrity and uniformity. The key advantage of the die is its ability to preserve the integrity of the layers to the micro-nano scale. Furthermore, it is possible to increase the number of layers without prolonging the residence time, which is a crucial feature of this die design, especially when processing thermally sensitive materials. The new die was designed to seamlessly combine with existing cast coextrusion setups and was adapted into the coextrusion system described above.

**Figure 1:**
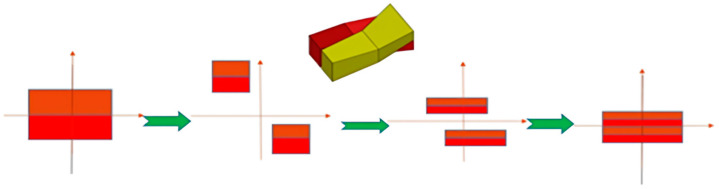
Illustration of the layer multiplication.

**Figure 2:**
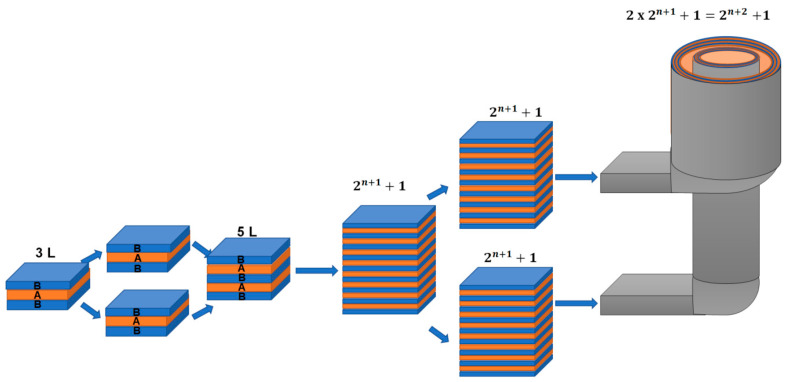
Illustration of the layer multiplication system and the novel blown coextrusion die for a Three-Layer BAB Film.

The process starts with a three-layer structure, including ethylene-vinyl alcohol (EVOH) as the internal layer and polyethylene (PE) as the external layers. As the structure moves through the multiplication system (Figure 1), a multilayer structure with 2^n+1^ + 1 layers is produced. Finally, the blown die is used to shape this refined structure into its final form. The multilayer structure can be multiplied without prolonging the residence time: the materials are simply entered into the blown coextrusion die head according to the illustration in Figure 2.

The modular design of the die enables a precise control of the flow, as it allows for the selection of the flow path in its entry by dividing the flow to the upper and the lower entry in order to multiply the number of layers. In addition, it provides the possibility of not further multiplying the structure by using only the upper or the lower entry. This flexibility guarantees better stability and layer integrity while also improving process control, and provides the upper flow with the capability to encapsulate the lower one. To guarantee the consistent film thickness of the bubble, adjustable screws are placed all the way around the circumference of the die to adjust the die gap by carefully tightening or loosening these screws.

#### 2.3.3. Multilayer Film Preparation

To assess the flow stability, the multilayer film development campaign started by creating films with a variety of architectures and compositions via cast coextrusion. Table 2 displays the temperature profile in the process. The temperature profile was selected based on the rheological properties of the materials, specifically, to prevent the degradation of the thermally sensitive EVOH and to achieve the best rheological match between the polymers, with viscosity and elasticity ratios close to 1.

Following the flow stability investigation, the novel blown coextrusion process was implemented to create the multilayer films. Table 3 lists the precise compositions with the various volume fractions.

Multilayer films were fabricated with 3, 129, and 1025 layers. Table 4 displays the nominal thickness of each EVOH layer for various percentages and numbers of layers.

(1) $$h_{\mathrm{nom}\;A}=h_{\mathrm{film}}\times \frac{\mathrm{vol}\%A}{n_{A\;\mathrm{layers}}}$$

- ℎ_nom A_ is the nominal thickness of the A material layer.
- ℎ_film_ is the total thickness of the multilayer film.
- vol%A is the volume percentage of A in the multilayer film.
- *n*_A layers_ is the number of A layers in the multilayer film.

## 3. Results and Discussion

### 3.1. Rheological Properties

The rheological behavior of polymers is a fundamental parameter when studying flow stability during coextrusion. In order to obtain stable films, the viscosity and elasticity ratios between the polymers must be optimized [30,39]. Understanding these properties is fundamental so as to define the processability window and ensure stability during the multilayer coextrusion process.

**Figure 3:**
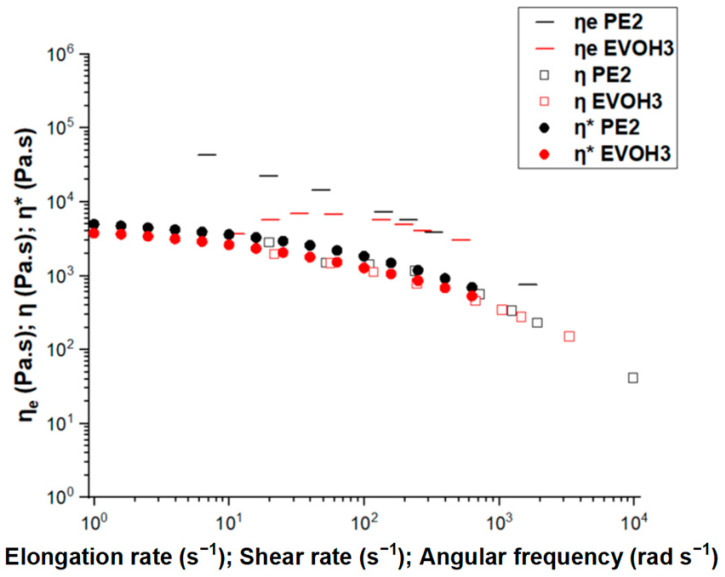
Complex, shear, and extensional viscosity of PE and EVOH.

The linear viscoelasticity (LVE) and high-shear-rate behavior of PE and EVOH, as determined through dynamic and capillary rheometry, and the elongation properties obtained by the Cogswell model are presented in Figure 3. The overlap of complex viscosity (η*) as a function of angular frequency (*ω*) and shear viscosity (*η*) as a function of shear rate ($\dot{\gamma }$) from capillary rheometry validates the Cox–Merz rule for both polymers.

(2) $$\eta \left(\dot{\gamma }=\omega \right)=\left|\eta *\right|\left(\omega \right)$$

Furthermore, the elongational viscosity η_E_ was evaluated by the Cogswell method [34]. The behavior suggests that PE has a strong resistance to elongational flow. When it comes to the extensional viscosity of EVOH, it has a much lower value compared to that of PE, thus indicating less resistance to elongation for EVOH. The next section presents a more thorough study of the extensional viscosity of the materials.

**Figure 4:**
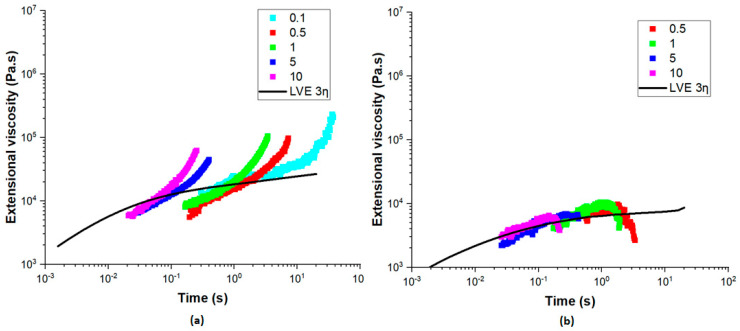
Extensional viscosity with the extensional rate varying from 0.1 to 10 s^−1^ for (**a**) PE at 170 °C and (**b**) EVOH at 210 °C.

Extensional rheology is a very important factor in polymer processing, particularly in film blowing and any processes involving elongational flow. Figure 4a represents the extensional viscosity of PE measured at Hencky strain rates between 0.1 and 10 s⁻^1^ at a temperature of 170 °C. It can be seen that PE exhibited prominent strain hardening, particularly at higher strain rates, where the extensional viscosity increased with time. The PE utilized in this study is linear low-density polyethylene (LLDPE), characterized by short-chain branching and a small amount of long-chain branching. This molecular architecture enhances strain hardening behavior, which is beneficial for bubble stability during the blown coextrusion process. The short-chain branches contribute to the polymer’s flexibility and toughness, while the limited long-chain branching provides sufficient entanglement to resist deformation under extensional stress. The alignment of PE’s extensional viscosity with the LVE envelope confirms that the material’s elongational behavior is consistent with its shear properties.

The extensional behavior of EVOH is, however, quite different, as shown in Figure 4b. With time, the extensional viscosity decreases in the case of different Hencky strain rates, presenting a strain-softening behavior. This would mean that EVOH is less resistant to the elongational flow and it less capable of supporting its structure under stretching as compared to PE. Such large discrepancies in rheological properties, namely the strong strain-hardening of PE and the strain-softening of EVOH, give grounds to challenges for maintaining stability during coextrusion.

### 3.2. Rheological Contrast and Flow Stability Investigation by Cast Coextrusion

Flow stability analysis in the coextrusion of multilayered structures is a fundamental tool to establish robustness and viability for processing. Flow stability was studied in the laboratory-built multilayer cast coextrusion setup with the goal of establishing the processability window. Several flow rate ratios and compositions were analyzed to obtain a stable multilayer coextrusion process. The first objective was to represent the coextruded multilayer films’ stability by using stability maps illustrating the operating conditions that would result in defect-free films.

**Figure 5:**
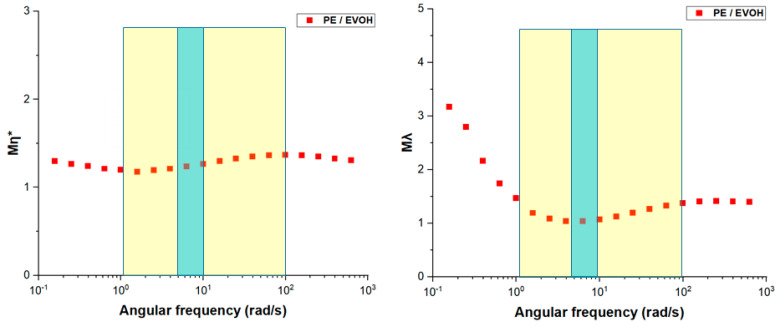
Viscosity and elasticity ratios of PE-EVOH at 200 °C, showing typical shear-rate ranges for extruders, and multiplier elements.

From dynamic rheological measurements, the ratios of viscosity (M_η_) and elasticity (M_λ_) for PE and EVOH were calculated. These ratios are crucial for evaluating interfacial stability, as rheological mismatches between layers can introduce defects. Figure 5 illustrates viscosity and elasticity ratios plotted versus the angular frequency, which is associated with the typical shear rates encountered during coextrusion. The yellow areas depict the range of shear rates typically observed in feedblocks and extruders (1–100 s^−1^), while the blue areas show the shear rates in the multiplier elements (1–10 s^−1^) [40,41]. Since viscosity and elasticity ratios of PE and EVOH remain close to one within the applicable shear rates of the coextrusion process, compatibility in the rheological perspective is assured. Thus, good interface stability should be expected for this combination.

To further investigate the flow stability during coextrusion, stability maps that represent the appearance and condition of the films as a function of the mass flow rates of the layers in contact were created. These flow ratios directly reflected the thickness ratios between the layers, helping to define the processability window for the material combination.

Based on the visual inspection of the coextruded films, the film characteristics that appeared in the stability maps were classified into three main groups:

- Stable: referring to films without any visible defects or instabilities.
- Wavy: regarding films with flow instabilities appearing as waves across the film surface.
- Unstable: describing films as having important defects.

**Figure 6:**
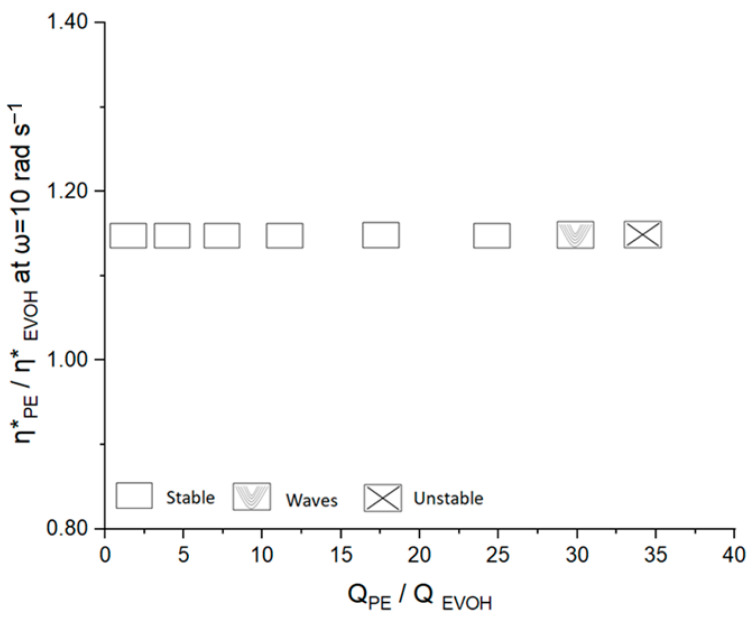
Stability map of a three-layer PE-EVOH film.

Figure 6 shows the stability map for a PE/EVOH/PE multilayer film and illustrates how stable, wavy, and fully unstable films developed for different flow rate ratios. The broad stability zone seen for a large range of flow ratios implied that the PE/EVOH combination was robust to flow instabilities. To this effect, the production of stable multilayer films over a wide range of extrusion conditions was deemed possible.

**Figure 7:**
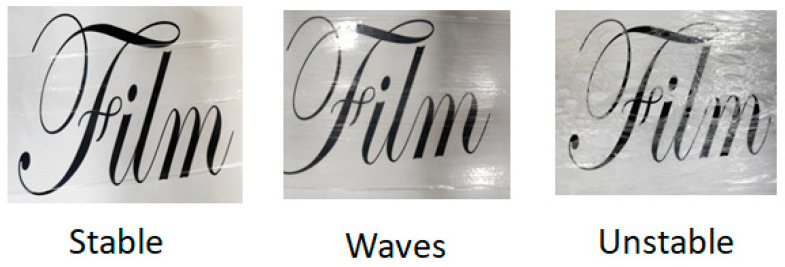
Photographs of multilayer films: visualization of a stable film, wave-like instabilities, and the presence of defects.

To visually represent the different types of flow instabilities that can occur during coextrusion, images of the coextruded films are presented in Figure 7. The comparison between the photographs and the stability maps demonstrated the critical importance of achieving a balanced rheological profile and carefully controlling the mass flow ratios during coextrusion. Maintaining stable flow conditions is essential in order to produce defect-free multilayer films, while deviations in flow ratios can lead to the development of wave-like instabilities or more severe film defects.

The flow stability investigation provided a solid foundation for optimizing the multilayer coextrusion process, particularly in view of scaling up to more complex architectures or transitioning to different coextrusion die technologies, such as blown coextrusion.

### 3.3. Stability Investigation of Blown Coextrusion

The elaboration of multilayer films by the blown coextrusion process requires a careful optimization of both material properties and processing parameters. This section discusses an investigation of the stability of multilayer films comprised of PE and EVOH; particularly, how the content of EVOH and the number of layers influence the stability of the process. A stability chart is a graphical representation defining the limits of the processability window. The stable zones correspond to those regions that define the defect-free conditions of processing, while the unstable zones correspond to the region where film defects or instabilities can be expected.

In blown film coextrusion, there are two important parameters that are used to assess the process stability: the Blow-Up Ratio (BUR) and the Take-Up Ratio (TUR). The BUR is defined as the ratio of the diameter of the bubble to the diameter of the die, describing the degree of expansion of the film in the transverse direction. The TUR, on the other hand, represents the ratio between the velocity of the film and the melt velocity, which gives the quantity of stretching, applied in the machine direction. Both ratios play a significant role when it comes to bubble stability and uniformity of the film properties, since a deviation from optimum BUR and TUR values can lead to film rupture, non-uniform thicknesses, or surface defects.

**Figure 8:**
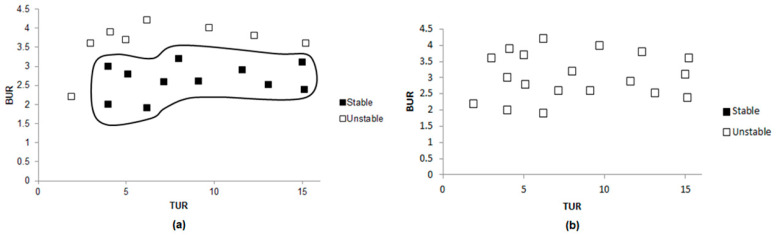
Stability charts for (**a**) PE-1L film and (**b**) EVOH-1L film.

The present work targeted several goals, the first of which was to establish some baseline stability data for single-layer films of PE and EVOH. Figure 8a shows a stability chart for single-layer polyethylene (PE-1L) film, indicating that PE has a large processability window, as significant portions of the map contained black squares where the film can be processed without defects. Stability can be observed in a wide range of values for both BUR and TUR. In contrast, the stability chart for a single-layer EVOH film, shown in Figure 8b, presents no areas of stability, as indicated by the fact that no black squares were present. The absence of stable regions can be attributed to EVOH’s inherent rigidity and limited flexibility.

**Figure 9:**
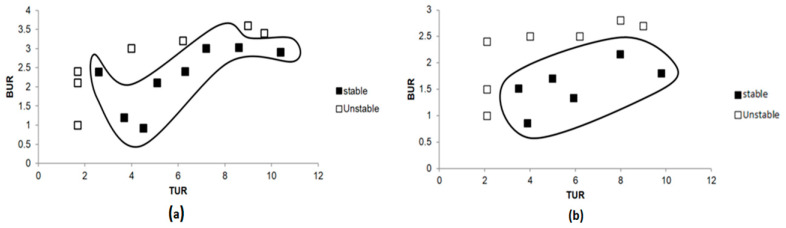
Stability charts of 129-layer films with different percentages of EVOH: (**a**) 4% of EVOH and (**b**) 12% of EVOH.

Following the single-layer film evaluation, multilayer films with varying EVOH content were studied. Films with 129 layers were fabricated, in which the EVOH content was either 4%, 8%, or 12%. The objective was to explore the effect of the composition on the processing stability. Films with 4% of EVOH presented a fairly large area of stability (Figure 9a). This stability was most pronounced at moderate values of both BUR and TUR, which would indicate that the film could be processed defect-free within a wide processing window. Increasing the EVOH content to 12% resulted in a significant decrease in the area of stable processing, as can be seen by the stability map in Figure 9b. With fewer black squares, this composition was quite prone to instability. Both high and low values of the BUR increase the probability of film defects, since the EVOH layers become stiffer.

**Figure 10:**
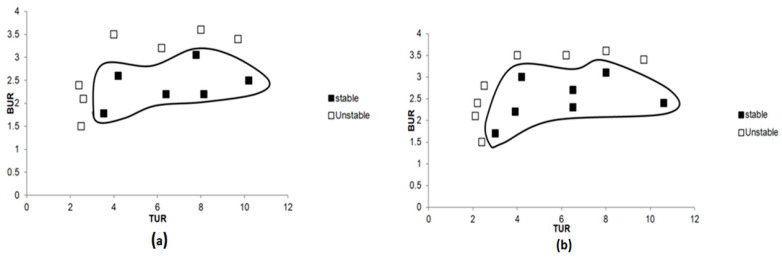
Stability charts of films with 8% of EVOH and different numbers of layers: (**a**) 3 layers and (**b**) 1025 layers.

Multilayer films were also produced with 8% of EVOH. PE-EVOH8% with three layers (Figure 10a) presented a narrow range of stability. Thus, with only three layers, the structural integrity was low, and the film was more prone to defects. Figure 10b represents the stability of PE-EVOH8% with a 1025-layer configuration, showing a considerable increase in stability. Indeed, this structure exhibited the widest processability window with the least number of instabilities over a wide range of values for both BUR and TUR. In such a configuration integrating a large amount of layers, an added structural integrity is developed within the film, thereby allowing defect-free films to be reproduced consistently. Results have shown that the increase in the number of layers leads to a great stability improvement in the process of blown coextrusion, with the 1025-layer film achieving the best overall stability.

**Figure 11:**
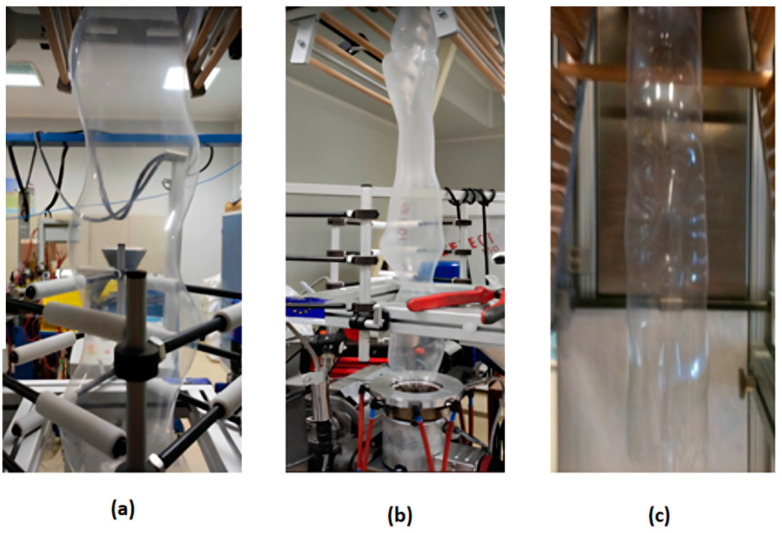
Instabilities observed during blown coextrusion: (**a**) Dancing effect. (**b**) Helical instability. (**c**) Breathing effect.

In addition to the stability charts presented above, the visual appearance of the films with a few of the most common defects in the unstable regions of the stability charts is shown in Figure 11. The most common among them, known as the dancing defect, is characterized by a rhythmic lateral movement of the bubble. This defect was quite prominent in the films with higher EVOH content and was most pronounced at low BUR values. Another observed defect was helical instability, which is a kind of spiral deformation of the film bubble. This generally occurred at intermediate values of BUR with high TUR. Finally, a breathing defect, recognizable by its periodic fluctuations, was also noticed.

In summary, the stability of blown coextruded PE-EVOH multilayer films was influenced by the content of EVOH and the number of layers. Films with the lowest EVOH content and the largest number of layers showed the greatest processability window, in which defect-free films could be fabricated within a large range of BUR and TUR. Conversely, films with a higher EVOH percentage and fewer number of layers had a much more reduced stability.

### 3.4. Transmission Electron Microscopy (TEM) Analysis of Multilayer Films

**Figure 12:**
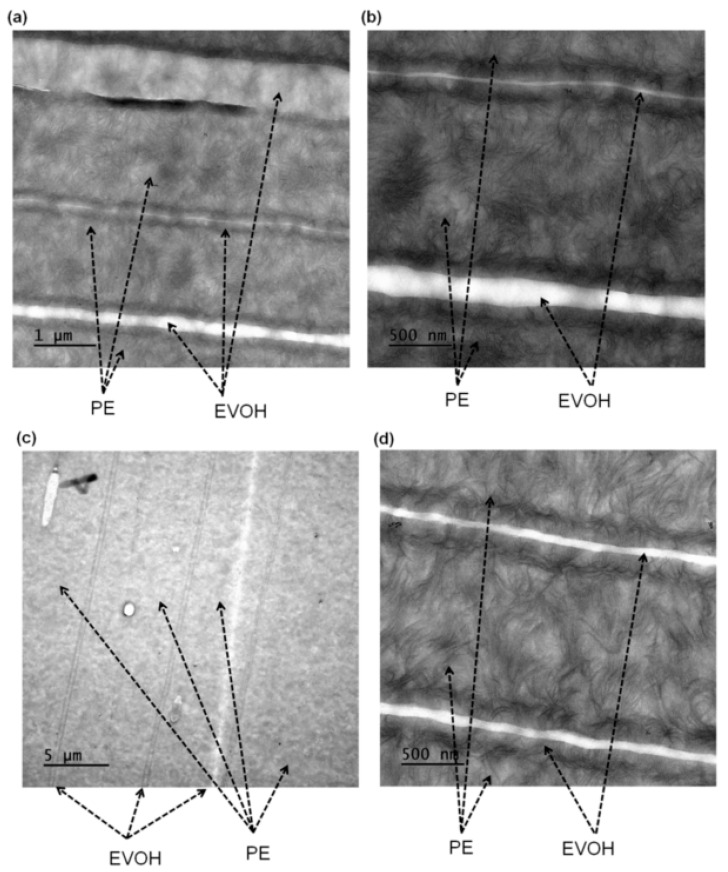
TEM micrographs of PE-EVOH12% multilayer films: (**a**) 129-layer film at low magnification, (**b**) 129-layer film at higher magnification, (**c**) 1025-layer film at low magnification, and (**d**) 1025-layer film at higher magnification.

TEM characterizations provided a clear insight into the complex structural features in PE-EVOH multilayer films. TEM micrographs of 129- and 1025-layer films with 12% EVOH are presented in Figure 12. For the 129-layer film, the images revealed well-defined thin EVOH layers distributed within the PE matrix (Figure 12a,b). These continuous and well-spaced EVOH layers demonstrated clear boundaries between the two kinds of polymers, indicating successful processing. Furthermore, Figure 12c,d illustrates the PE-EVOH 12% 1025-layer film. The much higher number of layers can be clearly observed. Thinner and closer to each other, the layers of EVOH caused an increase in the density of the interfaces. The preservation of nano-scale layer structures reflects the precision of the process in producing films with a high number of layers and consistent layer thicknesses.

### 3.5. Extensional Rheology of Multilayer Films

**Figure 13:**
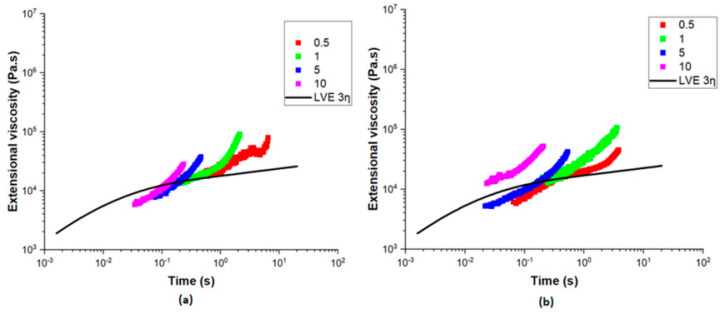
Extensional viscosity versus extensional rate of multilayer films: (**a**) PE-EVOH4% 3 L and (**b**) PE-EVOH12% 3 L.

**Figure 14:**
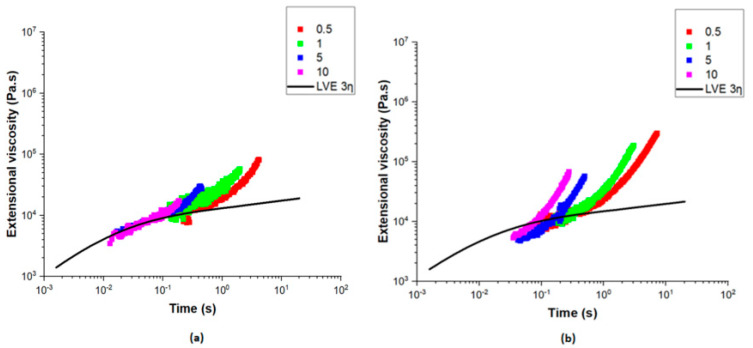
Extensional viscosity versus the extensional rate of multilayer films: (**a**) PE-EVOH8% 3 L and (**b**) PE-EVOH8% 1025 L.

The extensional viscosity of the PE-EVOH multilayer films, with varying percentages of EVOH (4%, 8%, and 12%) and different layer configurations (3 L and 1025 L), was measured at 200 °C and is plotted in Figure 13 and Figure 14 with their linear viscoelastic envelope (LVE). The data show the influence of the EVOH content in three-layer films. Differences in strain-hardening behavior were observed, especially at higher extensional rates. These were, however, not pronounced enough to imply a leading role of the EVOH content in influencing the film’s resistance towards elongation. The strain-hardening behavior of the PE-EVOH4% 3 L film was moderate, while the PE-EVOH12% 3 L film had weaker strain-hardening behavior.

In contrast, while studying films with 8% EVOH (Figure 14), the strain hardening was observed to be very sensitive to the number of layers. For instance, the 1025-layer film exhibited significantly greater extensional viscosity, particularly at the higher Hencky strain rates, than its 3-layer counterpart. The strong enhancement in strain hardening, attributed to the contribution of the interfaces, suggests that increasing the number of layers leads to an enhanced distribution of stress within the film. The improved strain hardening was directly related to the processing stability when increasing the layer count. For example, the 1025-layer film demonstrated a much higher resistance to deformation, as evidenced by the rise in extensional viscosity at higher strain rates.

The above-mentioned findings were in agreement with the larger processability windows observed in the stability charts, where films composed of more layers showed a wider stability. Films with fewer layers such as the three-layer films tend to show reduced strain hardening and, therefore, more instability, as evidenced by a narrower stability.

## 4. Conclusions

The present study addresses a critical absence in the literature on blown coextrusion through the development and validation of a novel die technology. Unlike conventional blown dies, the presented modular system maintains layer integrity in the micro-nano scale. This new die design can be seamlessly combined with existing cast coextrusion setups, and renders it possible to increase the number of layers without increasing the residence time, since there is no need for an additional multiplier element.

Rheology is a main actor in this innovation, especially in the investigation of flow stability. In the present work, the rheological properties were related to stability charts, establishing the process stability in coextrusion. It was demonstrated that an increase in the number of layers widened the processability window. It should be mentioned that the multilayer films exhibited good adhesion between layers without the need of tie layers, thus simplifying the film structure and improving recyclability, which is a major advantage for sustainability and a contribution to the circular economy.

The transparency of the multilayer films was also preserved at high levels of EVOH, ensuring that they met the optical demands of packaging applications. In conclusion, the present work expands the capability of blown coextrusion for multilayer films, presenting significant advances in processing stability, material efficiency, and sustainability. Detailed studies on barrier properties will be addressed in a future publication. The new die design, coupled with a profound understanding of the relationship between rheology and processability, opens new paths toward the development of cost-effective high-performance packaging materials.

## Figures and Tables

**Table 1:** Description of the used polymers.

Abbreviation	Material	Manufacturer Name	Density (g/cm^3^)	MFR (g/10 min)	MFR Test Conditions
PE	LLDPE	Marlex^®^ D139	0.918	1	190 °C/2.16 kg
EVOH	EVOH	EVAL™ F101B	1.19	1.16	190 °C/2.16 kg

**Table 2:** Temperature profile of the coextrusion process.

Temperature Profile (°C)				
Extruder Zone 1	Extruder Zone 2	Extruder Zone 3	Feedblock	Die
220	220	220	210	200

**Table 3:** Multilayer film compositions.

PE (%)	EVOH (%)
88	12
92	8
96	4

**Table 4:** Number of layers and nominal layer thickness of multilayer films obtained by blown coextrusion.

Number of Multiplier Elements	Total Number of Layers	h nom. EVOH 12% (nm)	h nom. EVOH 8% (nm)	h nom. EVOH 4% (nm)
0	3	18,000	12,000	6000
6	129	281	188	94
9	1025	35	23	12

## Data Availability

The data presented in this study are available on request from the corresponding author.
